# Real-Time Optimization of Discrete Element Models for Studying Asphalt Mixture Compaction Characteristics at the Meso-Scale †

**DOI:** 10.3390/s25030638

**Published:** 2025-01-22

**Authors:** Xue Wang, Zifang Wang, Xuanye Luo

**Affiliations:** 1 School of Highway, Chang’an University, Xi’an 710064, China; 2024221328@chd.edu.cn; 2 Chang’an Dublin International College of Transportation, Chang’an University, Xi’an 710064, China; 19560967985@163.com

**Keywords:** asphalt mixture, compaction, particle scale, discrete element model, Kalman filter

## Abstract

To enhance the quality of asphalt mixture compaction, the compaction mechanism, particularly at the meso-scale, must be thoroughly understood. Researchers have employed various technologies to study the compaction process. Among these, discrete element modeling (DEM) has been widely adopted due to its effectiveness in addressing particle-scale problems. However, improving simulation accuracy while maintaining computational efficiency remains a challenge. Therefore, this study aims to establish and optimize a DEM model for asphalt mixture gyratory compaction by integrating real-time laboratory SmartKli^®^ sensing data. The Kalman filter was implemented as the fusion algorithm to enable real-time calibration. Two SmartKli sensors were positioned at different locations within specimens during both the laboratory Superpave gyratory compaction (SGC) test and DEM simulation to investigate the particle rotation characteristics. The results showed that the upper layer exhibited a higher degree of compaction than the lower layer. After calibration, particle rotation in the optimized DEM model more closely matched the laboratory SmartKli sensing data. Additionally, the changing trends of relative rotation for both SmartKli particles and coarse aggregate particles improved. The SmartKli simulation ball effectively represented the rotational behavior of its surrounding coarse aggregates, making its kinematic responses a reliable predictor of asphalt mixture compaction characteristics at the meso-scale.

## 1. Introduction

The quality of asphalt mixture compaction has always been a major concern for field engineers because of its significant effects on material compactness and uniformity, as well as pavement’s performance in the long term. Compaction is defined as the process of asphalt mixture changing from a very loose state to a more coherent mass under external forces at the macro-scale [1]. In essence, the densification of the asphalt mixture was achieved through the continuous movement, rotation, and interaction of aggregate particles with the assistance of the lubricating asphalt binders at the meso-scale [2]. Thus, the studies focusing on asphalt mixture compaction are transforming from the macro to meso-scale, i.e., particle-scale, to illuminate the compaction mechanism. Various methods were developed and used to explore what happened inside the mixture during compaction and explain its relationship with the mixture’s macroscopic properties, which could greatly help with the compaction quality control process.

Laboratory compaction was commonly used because of its convenience in studying material properties. It mainly included impacting compaction, kneading compaction, gyratory compaction, and rolling wheel compaction with different working mechanisms. Among those methods, the Superpave gyratory compaction (SGC) is most commonly used. Researchers generally agreed that the SGC could apply shearing and compressive forces simultaneously to the mixture so that SGC could well simulate the field compaction [3]. However, the process of aggregate movement and migration to an interlocked skeleton under the compound gyratory forces had not been fully understood. To inspect the internal structure of an asphalt mixture, nondestructive methods have been adopted in recent years to replace the traditional destructive methods (such as cutting the core samples) due to their inefficiency. Currently, popular nondestructive methods can be divided into three categories, X-ray CT and imaging techniques, simulation methods (mainly including finite element modeling (FEM) and discrete element modeling (DEM)), and the newly developed smart sensors.

In early years, Masad et al. proposed systematic analytical methods to quantify the air void distribution, the aggregate orientation, the aggregate contact, etc., in asphalt mixtures using X-ray CT and imaging techniques [4,5,6]. The results revealed the characteristics of an asphalt mixture’s internal structure in both laboratory specimens and field cores. It was found that the distribution of aggregates and air voids was sensitive to the compaction force mode [4]. Compared with simulation methods, the process of laboratory experiments is relatively time-consuming and has low cost efficiency, especially when discussing the dynamic changes inside the asphalt mixture during the whole compaction process. Thus, the FEM and DEM methods were rapidly developed in studying the meso-scale material properties in asphalt mixture compaction. DEM can simulate particle movement behavior (translation and rotation) and interaction among particles; thus, it is commonly adopted to simulate the asphalt mixture compaction. Chen et al. [7,8] proposed the basic gyratory compaction and vibratory compaction DEM simulation model using open source language. The characteristics of air void distribution obtained in Chen’s research were consistent with Masad’s conclusion, which initially proved the reliability of the DEM simulation model. Further, Gong and Liu et al. [9,10] tracked particle motional behaviors under compactive forces and analyzed the effect of material and compaction parameters on them using the DEM model.

The newly developed SmartKli sensor has attracted researchers’ attention in recent years because of its high similarity with coarse aggregates. It was used to replace coarse aggregate in asphalt mixture compaction and showed good performance in tracking particle movement and exploring the compaction mechanism. Studies found that the relative rotation pattern of a particle was directly related to the compaction process in both laboratory and field compaction [11,12]. The experimental research using SmartKlis shed light on the intrinsic relationships between the particle responses and the compaction of an asphalt mixture at the meso-scale [13]. Two indicators based on the particle relative rotation curve, the relative rotation capacity, and average residual rotation were further proposed to evaluate the compaction workability and predict the compaction degree [14,15]. The SmartKli sensor was proven to be reliable, effective, and feasible in detecting the particle responses in asphalt mixture compaction.

The modeling method would directly affect the material responses derived in the DEM model. Thus, the improvement of DEM modeling accuracy, which ensures computing efficiency when simulating asphalt mixtures, has increasingly gained the attention of researchers in recent years. Researchers have proposed several methods, including X-ray CT scanning, Matlab data processing, and laser scanning, to simulate the 3D shape of aggregate particles in DEM [16,17,18], which has greatly enhanced the simulation accuracy. Other methods included reducing the total amount of particle balls and scaling down the material modulus, which improves the modeling feasibility and reliability [19,20]. The accurate prediction of particle-level responses in DEM is significant because it affects the single-particle motion, multiple-particle interactions, and, eventually, the compaction properties of the total asphalt mixture. However, the current research is limited and has hardly optimized the DEM model at the particle scale.

This study aims to establish an asphalt mixture SGC compaction DEM model that could be calibrated in real time at the meso-scale using laboratory SmartKli sensing data. The particle rotation characteristics at different locations were first analyzed through laboratory experiments and DEM simulations. The calibration effects would be evaluated by analyzing particle motion behavior, the representativeness of SmartKli for general coarse aggregates, and the prediction of compaction status.

## 2. Materials and Methods

### 2.1. Laboratory SGC Test

**Figure 1:**
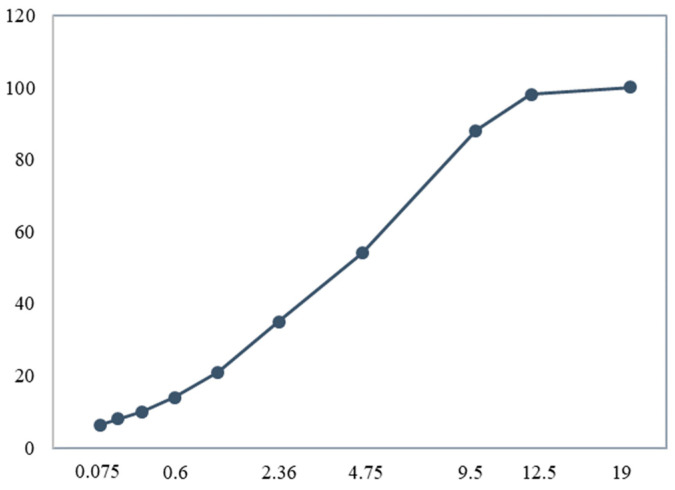
Asphalt concrete gradation chart.

**Figure 2:**
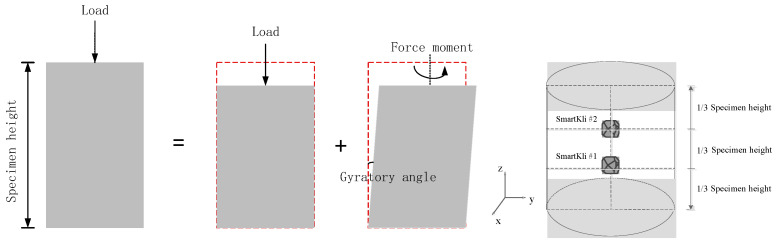
Laboratory experiment design: working mechanism of SGC and SmartKli embedment.

A type of commonly used dense-graded asphalt concrete (AC) material was used in the laboratory SGC compaction test. The mixture design and aggregate gradation information for AC are summarized in Table 1. The AC gradation chart is plotted in Figure 1. The working mechanism of the gyratory compactor used in this study is briefly demonstrated in Figure 2. The top plate applied vertical force to the asphalt mixture, and, at the same time, the mixture shearing forces were produced by the gyratory angle. The calibrated gyratory angle was 1.15°, the gyration speed was 30 rpm, and the compaction pressure was 600 Kpa. The designed air void of the compacted specimen was 4%.

**Figure 3:**
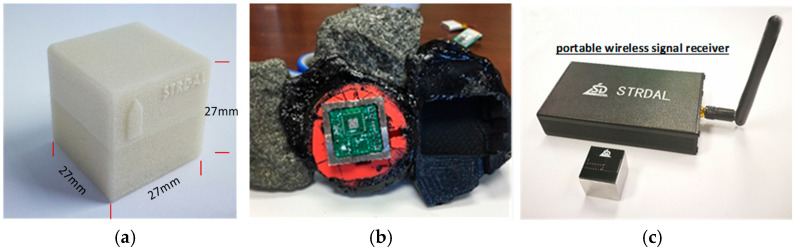
SmartKli sensor: (**a**) outside shell, (**b**) cohesion with asphalt materials, (**c**) signal receiver.

The wireless sensor, SmartKli, produced by the Sensor Technology Research Development and Application Laboratory (STRDAL) in Nanjing, China, was used during the asphalt mixture compaction to track the motions of internal aggregate particles. The SmartKli used in this study is a cube with a 27 mm edge length and a high strength, high-temperature resistant external shell, as shown in Figure 3. The SmartKli shell was designed as a cube in order to minimize its volume and decrease its disturbance to the compaction process of the asphalt material. The shell material was acrylonitrile butadiene styrene (ABS) with Nylon, which was found to have good compatibility and cohesion with asphalt material, as shown in Figure 3b. The SmartKli is equipped with a tri-axial gyroscope, a tri-axial accelerometer, and a tri-axial magnetometer, which can record rotation, translation, and orientation with nine degrees of freedom. The data can be wirelessly transferred to a computer based on BLE technology (location identification algorithm based on Bluetooth low-energy technology) in real time. Each SmartRock sensor has its own built-in global coordinate system, which is known to the researcher (the user). No matter how the SmartRock is placed in the structure, we can precisely track its orientation. The outside shape and the sensor’s placement do not affect the data collection and orientation determination of the final results [11]. In recent years, the SmartKli sensor has gained more attention for its superiority in detecting the kinematics of the infrastructure materials. It has been used to monitor particle movements in asphalt mixtures to investigate the compaction mechanisms [2,3], to detect material deformation and calculate material parameters [21,22,23], and to understand the service status of asphalt pavement structures [24,25]. The SmartKli sensor has demonstrated adequate convenience, reliability, and accuracy in reacting as a real coarse aggregate and recording particle responses under external loads.

In this study, two SmartKlis were embedded inside the specimen to simultaneously monitor the compaction process, and they were set at 1/3 and 2/3 of the depth layer along the centerline, as shown in Figure 2. SmartKlis were embedded in the 150 mm diameter mold, and the specimen was compacted to a height of approximately 170 mm, which could minimize the interference of the SmartKlis to the compaction process, protect the SmartKlis from boundary effects, and ensure data stability. Also, the selected locations in this study could represent different layers inside the asphalt mixture. One-third of the asphalt mixture was put into the mold first; SmartKli #1 was then set on the surface, and another one-third of the mixture was placed in the mold with SmartKli #2 located on top of it. The remaining third of the mixture was placed in the mold last. Three or more SmartKlis could be considered in future studies once the size of the sensors is reduced.

### 2.2. DEM Simulation of SGC Test

**Figure 4:**
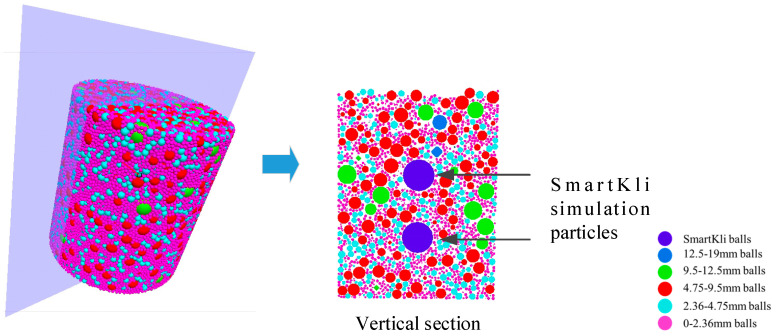
DEM simulation with two SmartKlis in SGC specimen.

The Particle Flow 3D (PFC3D) commercial DEM program from Itasca [26] was used in this study to simulate the SGC compaction process of the asphalt mixture. The simulation process included three main steps, the generation of the compaction mold wall and asphalt mixture particle balls, the exertion of gyratory and compressive forces, and the recording of simulation outputs. The particle balls were strictly generated according to the asphalt mixture’s gradation design, and the SmartKli simulation particles were generated and located at their designed locations, as shown in Figure 4. The vertical pressure of the compaction plate and the angle of gyration were set the same as in laboratory experiments, 600 KPa and 1.15°.

This study adopted ball elements to represent the aggregates, and the effects of particle angularity and surface roughness were indirectly considered through contact model parameter assignment. The method of using spherical balls to simulate asphalt mixture aggregates is commonly used by researchers because of its relatively high computing efficiency and simultaneous accuracy. In addition, the accurate simulation of particle shape, angularity, and surface texture complicates the nature of compaction [27]. Thus, spherical particles were adopted, and particle angularity and surface roughness were considered by adjusting Burger’s model parameters. The fine aggregates in the asphalt mixture would significantly increase the computing time, especially for the dense-graded asphalt concrete, as discussed in this study. Thus, fine aggregates with a diameter smaller than 2.36 mm and asphalt binder were considered asphalt mastic and simulated uniformly as 2 mm diameter balls. The two SmartKlis were simulated as 20 mm diameter balls since the previous study showed that the change in SmartKli ball size would not significantly affect the particle responses and compaction process in DEM [2]. The diameter of the specimen cylinder was set as 100 mm, which is acceptable because the aggregates were strictly generated according to their gradation, and the maximum aggregate size was smaller than one-third of the sample’s minimum dimension. Lastly, the mold gyration speed needed to be accelerated by a scale factor in order to rotate the container efficiently, considering the large number of ball elements. Thus, a damp ratio was added to the contact between particles to reduce the effects of accelerated gyration on particle movement. The detailed DEM modeling steps and explanation of the assumptions can be found in our previous study [2].

**Figure 5:**
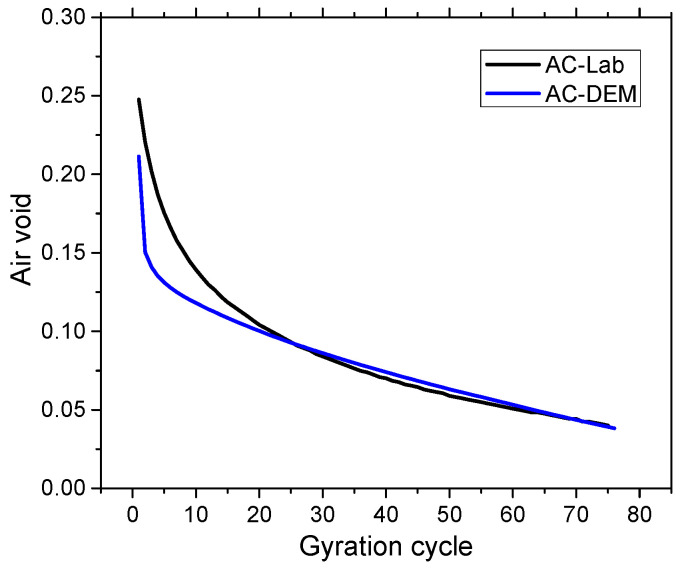
Mixture air void in laboratory test and DEM simulation.

Burger’s model was used at the ball’s contact to characterize the time-dependent viscoelastic behaviors of the asphalt mixture. The eight microscopic parameters K_kn_, K_ks_, C_kn_, C_ks_ K_mn_, K_ms_, C_mn_, and C_ms_ were used to describe the contact relations between two microscopic particles, as shown in Table 2, and were calculated with the macroscale parameters measured in the laboratory. The macroscale parameters represent material properties of the mastics and aggregates measured in the laboratory and were correlated to the experimental dynamic modulus and phase angle. The change in air voids during SGC compaction is an important property of asphalt mixtures, which can be directly affected by the mixtures’ packing and contact behaviors. Thus, the air void was used to calibrate the microscopic parameters of Burger’s model. The calibration process was realized by first conducting a sensitivity analysis based on a simplified model to capture the influence of each parameter on air void change. Only coarse aggregates were simulated in this simplified model to enhance the computing efficiency. Then, the initially assigned model parameters were adjusted to match the air void change in the simulation to the laboratory. The air voids of the AC mixture under the laboratory compaction and DEM simulation were plotted in Figure 5. Generally, the trend in air void change in the lab and DEM model were in good agreement, suggesting that the developed DEM model was reasonable. The result of the calibrated parameters is summarized in Table 3.

### 2.3. Real-Time Calibration of Virtual SGC Model Using Laboratory Test Results

**Figure 6:**
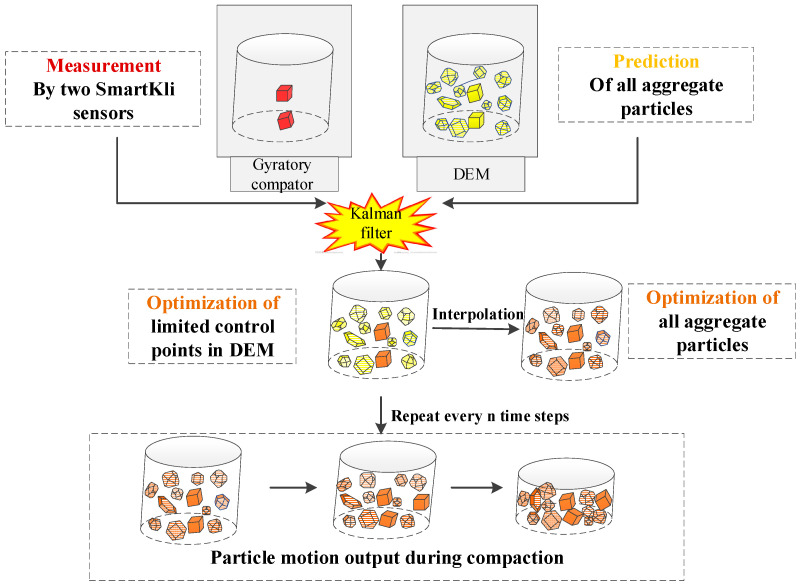
Schematic diagram of the real-time calibration process.

The real-time calibration of the virtual SGC model could be realized in the following four steps, as shown in Figure 6: (1) determination of SmartKlis’ rotation status at each time step from both the laboratory SmartKli sensor measurements and the DEM prediction results; (2) data fusion of sensor measurements and DEM prediction results using Kalman filter to optimize the rotation status of the SmartKli simulation balls in DEM; (3) optimization of the rotation status of other aggregate balls in DEM; (4) repeat of optimization process every n time steps to obtain the particle motion data during the whole compaction process.

#### 2.3.1. Rotation Status

In the virtual model, $\theta_{x}$, $\theta_{y}$, $\theta_{z}$, $\omega_{x}$, $\omega_{y}$, $\omega_{z}$, ${\dot{\omega }}_{x}$, ${\dot{\omega }}_{y}$, ${\dot{\omega }}_{z}$ were used to represent the ball’s angular rotation, angular velocity, and angular acceleration in the x-direction, y-direction, and z-direction. The ball’s rotation status at the k + 1th time step can be expressed with its status at the kth time step as shown in Equation (1) [28]:

(1) θωk+1=Aθωk+Bω˙+wk,

where

$$A=\left[\begin{array}{cccccc} 1 & 0 & 0 & ∆T & 0 & 0\\ 0 & 1 & 0 & 0 & ∆T & 0\\ 0 & 0 & 1 & 0 & 0 & ∆T\\ 0 & 0 & 0 & 1 & 0 & 0\\ 0 & 0 & 0 & 0 & 1 & 0\\ 0 & 0 & 0 & 0 & 0 & 1 \end{array}\right]\;\;\;\;\;\;\;\;\;\;\;\;\;\;\;\;B=\left[\begin{array}{ccc} \frac{1}{2}{∆T}^{2} & 0 & 0\\ 0 & \frac{1}{2}{∆T}^{2} & 0\\ 0 & 0 & \frac{1}{2}{∆T}^{2}\\ ∆T & 0 & 0\\ 0 & ∆T & 0\\ 0 & 0 & ∆T \end{array}\right]$$

$$\theta =[\theta_{x};{\;\theta }_{y};{\;\theta }_{z}]\;\;\;\;\;\;\omega =[\omega_{x};\omega_{y};\;\omega_{z}]\;\;\;\;\;\;\dot{\omega }=[{\dot{\omega }}_{x};\;{\dot{\omega }}_{y};\;{\dot{\omega }}_{z}]$$

∆T is the time step length, and the variable $w_{k}$ is assumed Gaussian noise with known covariance $E\left[\omega_{k}\cdot \omega_{k}^{T}\right]=Q$, where $E\left[\cdot \right]$ denotes the mathematic expectation.

In the laboratory, the rotation status of SmartKli at the kth time step can be denoted as shown in Equation (2):

(2) $$meass_{k}=s_{k}+z_{k}$$

where $meass_{k}$ represents the measurements of particle rotation at three axes through SmartKli sensing, $z_{k}$ is the Gaussian noise of angular displacement and angular velocity with covariance $E\left[z_{k}\cdot z_{k}^{T}\right]=R$. $\sigma_{x}^{2}$, $\sigma_{y}^{2}$, $\sigma_{z}^{2}$ denote the variance in particle rotation displacement, $\sigma_{\dot{x}}^{2}$, $\sigma_{\dot{y}}^{2}$, $\sigma_{\dot{z}}^{2}$ denote the variance in particle rotation velocity, and $\sigma_{x\dot{x}}$, $\sigma_{y\dot{y}}$, $\sigma_{z\dot{z}}$ denote the covariance in particle rotation displacement and velocity.

$$\sigma_{xy}=\sigma_{xz}=\sigma_{yz}=0\;\;\;\;\sigma_{\dot{x}\dot{y}}=\sigma_{\dot{x}\dot{z}}=\sigma_{\dot{y}\dot{z}}=0\;\;\;\;\sigma_{x\dot{y}}=\sigma_{x\dot{z}}=\sigma_{y\dot{x}}=\sigma_{y\dot{z}}=\sigma_{z\dot{x}}=\sigma_{z\dot{y}}=0$$

$$R=\left[\begin{array}{cccccc} \sigma_{x}^{2} & 0 & 0 & \sigma_{x\dot{x}} & 0 & 0\\ 0 & \sigma_{y}^{2} & 0 & 0 & \sigma_{y\dot{y}} & 0\\ 0 & 0 & \sigma_{z}^{2} & 0 & 0 & \sigma_{z\dot{z}}\\ \sigma_{x\dot{x}} & 0 & 0 & \sigma_{\dot{x}}^{2} & 0 & 0\\ 0 & \sigma_{y\dot{y}} & 0 & 0 & \sigma_{\dot{y}}^{2} & 0\\ 0 & 0 & \sigma_{z\dot{z}} & 0 & 0 & \sigma_{\dot{z}}^{2} \end{array}\right]$$

#### 2.3.2. Data Fusion

The rotation status of the SmartKli simulation ball in the virtual model was optimized through data fusion of sensor measurements and DEM prediction values. The data fusion algorithm was realized in seven steps:

(1) Acquire the ball’s rotation status θωk at the kth time step in DEM.
(2) Import the measurements of SmartKlis into the virtual model.
(3) Calculate the difference between the SmartKli measured value and the SmartKli simulation ball predicted value:

(3) ∆k=meassk−θωk

(4) Update the ball’s rotation status at the k+1th time step using the Kalman filter. The mathematical description of the Kalman filter is shown in Equations (4)–(8) [29]:

(4) $${\hat{x}}_{\bar{k}}=A{\hat{x}}_{k-1}+Bu_{k-1}$$

(5) $$P_{\bar{k}}=AP_{k-1}A^{T}+Q$$

(6) $$K_{k}=\frac{P_{\bar{k}}H^{T}}{HP_{\bar{k}}H^{T}+R}$$

(7) $${\hat{x}}_{k}={\hat{x}}_{\bar{k}}+K_{k}({meass}_{k}-H{\hat{x}}_{\bar{k}})$$

(8) $$P_{k}=(I-K_{k}H)\;P_{\bar{k}}$$

where $K_{k}$ = the Kalman gain for particle rotation that gives relative weight to measurements and DEM prediction; $P_{k}$ = the results of current state covariance matrices; $P_{\bar{k}}$ = the prediction state covariance matrices; H = 6 × 6 identity matrices; ${\hat{x}}_{\bar{k}}$ = the prediction state matrices based on the current rotation status; ${\hat{x}}_{k}$ = the results of current state matrices with respect to particle rotation. R is the covariance matrices calculated from the noise of SmartKli measurements. Q is determined based on experience. $P_{0}$ is initialized as 1 because of its limited effects on the optimization in the cyclic updating process [28].

#### 2.3.3. Optimization of All Particles During Compaction

In the laboratory test, only a limited number of coarse aggregates can be replaced by SmartKli sensors to measure their motion status. Thus, the data fusion and optimization process of other aggregate particles should rely on the estimated ${∆}_{k}$ values. When regarding the SmartKlis as control nodes, the ${∆}_{k}$ of other aggregate particles could be described with the IDW (inverse distance weighting) method, which means that particles closer to the control node i would be assigned with greater weight of node i’s ${∆}_{k}$ value. Liu has proved that the Shepard interpolation method could provide fast interpolation with reasonable accuracy and can be implemented in PFC3D without massive matrix operations [28]. Thus, the Shepard interpolation method was adopted in this study to obtain the distribution of ${∆}_{k}$.

This optimization frequency should be kept consistent with the SmartKli data collection frequency. Therefore, the above data fusion and calibration process was evoked 16 times per second during the DEM simulation process.

## 3. Results and Discussions

### 3.1. Characterization of Particle Rotation at Different Locations in SGC

#### 3.1.1. Measurements of Particle Rotation in Laboratory Tests

**Figure 7:**
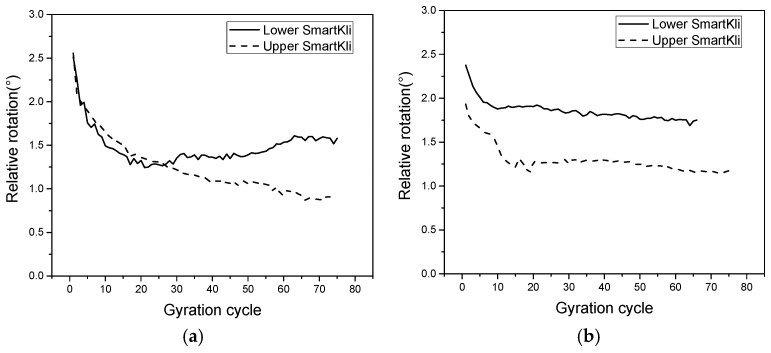
Relative rotation of SmartKlis at different locations in SGC: (**a**,**b**) the results from two repetition tests.

The particle’s relative rotation is defined as the difference between the peak and the valley values of the rotation angle for each gyration cycle, and it was correlated with the asphalt mixture’s density change during gyratory compaction [11]. In this study, the particle’s relative rotation was measured by SmartKlis at different locations in two repeated SGC tests and plotted in Figure 7a,b. As shown in the figure, generally, the relative rotation of the particles showed a decreasing trend in the beginning that stabilized in the later compaction stage, which was related to the mixture’s densification process. However, the particles in the upper location tended to have smaller relative rotation values and needed more gyration cycles to stabilize compared with particles in the lower location. This phenomenon was related to the asphalt mixture’s compaction mechanism in SGC. The compactive force is transmitted from the top compressive plate to the bottom of the specimen; thus, the upper part undertakes more compaction effects for a longer time, and more compactive energy is transformed to the aggregates’ kinematic energy in the upper part of the specimen. Therefore, the upper particles would take more gyration cycles to rotate and adjust their position before stabilization. Additionally, the relative rotation of the upper particles is relatively small due to the greater compaction degree in the upper layer. The differences between the two repeated tests could be produced by environmental noises, inherent mechanical errors in the sensors, and material heterogeneity, which needs more tests and further studies to enhance data stability.

#### 3.1.2. Prediction of Particle Motion in DEM

**Figure 8:**
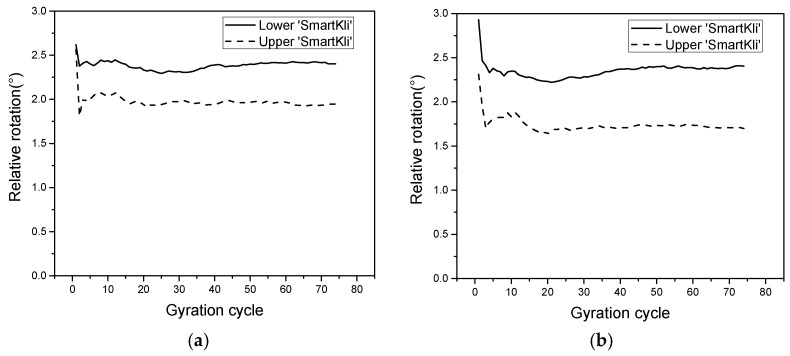
Relative rotation of SmartKlis simulation balls at different locations in DEM: (**a**,**b**) the results from two repetition tests.

The relative rotation of the two SmartKli simulation balls in two repeated DEM tests was calculated and presented in Figure 8a,b. Figure 8 denoted that the overall changing trend of the particle’s relative rotation was the same as the laboratory test results. Particles would have a higher relative rotation value at the start of compaction and then decrease to a stabilized value in the later stage. In addition, the relative rotation of the lower SmartKli ball was smaller than that of the upper SmartKli ball, which was consistent with the laboratory measurements. Compared with the laboratory test results shown in Figure 7, the particle’s relative rotation in DEM was larger on the whole, and the particle would enter the stabilized stage much earlier. The summary of transition cycle N is shown in Table 4 (the transition cycle is defined as the gyration cycle N at which the decreasing trend of the particle’s relative rotation transforms from a rapid to a smooth status; for the calculation of N, refer to [2]). The particle would enter a stabilized stage in only 3 to 4 cycles in DEM, and the difference between the upper and lower particles was not obvious. This changing trend was not reasonable since the compaction was a gradually working process that could not be sharply accomplished in the first few gyration cycles.

**Figure 9:**
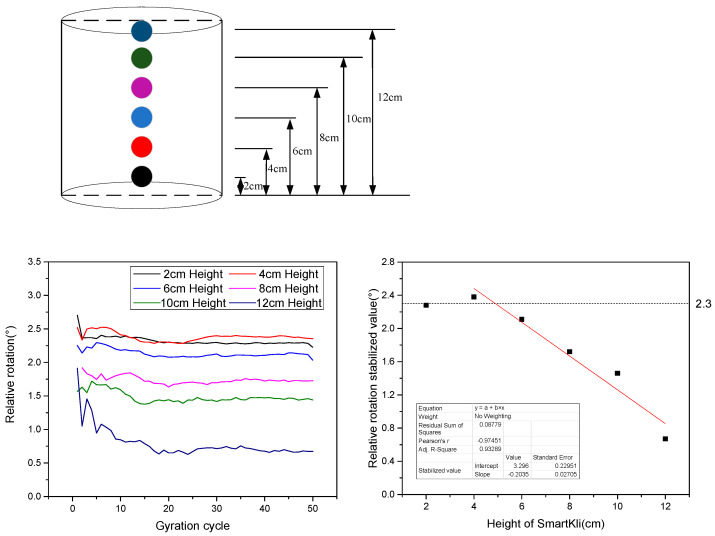
DEM simulation with SmartKli particle designed at different vertical locations and their relative rotation results.

To further investigate the particle’s motion characteristics at different vertical positions in gyratory compaction, the DEM simulation was conducted with SmartKli simulation balls settled at different vertical positions, as shown in Figure 9. The relative rotation curves collected by the SmartKli ball in the simulation tests are plotted in Figure 9. In general, particles in a higher layer tended to have smaller relative rotational values. It can be seen that the stabilized relative rotational value of particles higher than 4 cm decreased with increasing location height in an approximately linear way. The adjusted R square of the fit line was larger than 0.9. However, particles lower than 4 cm had consistently stabilized relative rotation values of 2.3°, which was mainly determined by the compactor’s working mechanism. The kneading effect of gyratory compaction was realized by rotating the mold around its top center and tilting its bottom part. Thus, the stabilized relative rotation angle would increase following the decreasing particle height until it reached 2.3°.

The particle’s relative rotation trend presented by DEM generally agreed with the laboratory-measured results. The DEM simulation was more convenient for obtaining particle motions than the laboratory test. However, due to the different monitoring mechanisms, the particle rotation responses detected in the laboratory test and DEM simulation were still different. Thus, it is essential to optimize the DEM simulation results at the particle scale.

### 3.2. Evaluation of the Optimization Effect

#### 3.2.1. Optimization of the SmartKli Particle Motion

**Figure 10:**
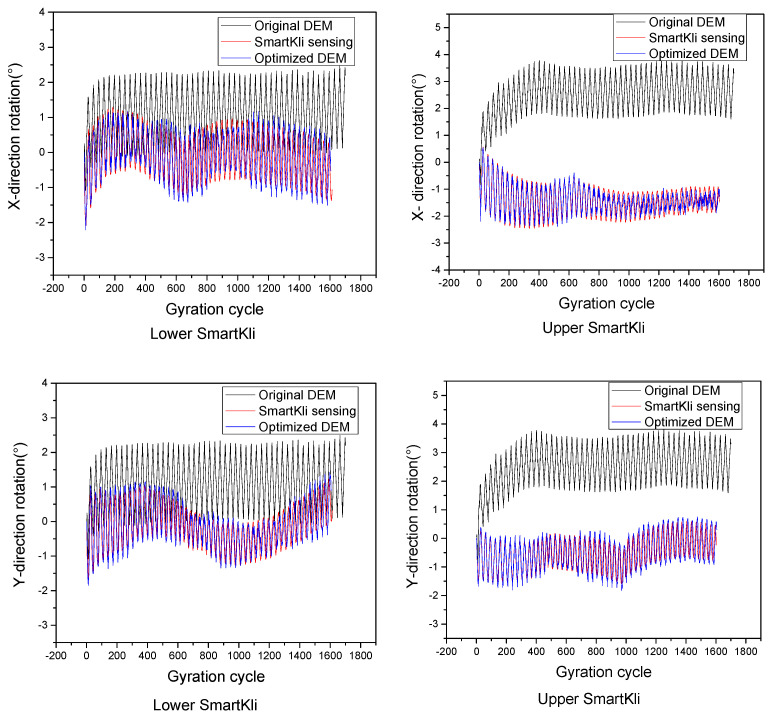
Optimization effects presented by particle’s rotation.

**Figure 11:**
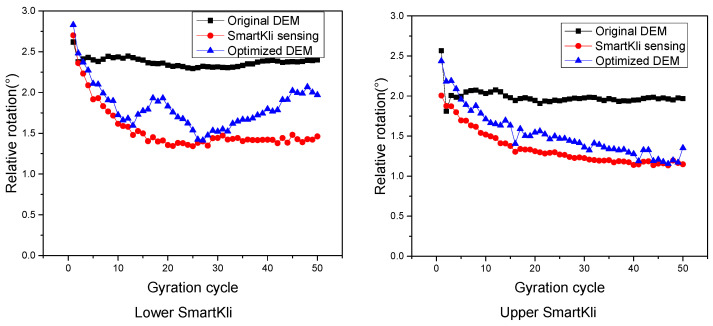
Optimization effects presented by particle’s relative rotation.

The particle rotation predicted in the original virtual SGC model was calibrated using the method introduced in Section 2.3. The particle rotation responses of the two SmartKlis, as measured by SmartKli sensors, predicted in the original DEM model and the optimized DEM model of trial 1, are plotted in Figure 10. It can be seen that the SmartKli particle’s rotation status changed in the optimized DEM model. The optimized particle rotation simulation results moved closer to the laboratory SmartKli sensing results. The particle’s relative rotation was further calculated and is presented in Figure 11. It can be seen that its changing curve after optimization was located between the SmartKli measured values and the original DEM-predicted values. Most importantly, the change in the particle’s relative rotation trend was improved in the optimized DEM model. It would gradually decrease in the early compaction stage before stabilization instead of decreasing sharply at the first three to four cycles, as presented in the original DEM model.

#### 3.2.2. Optimization of the Coarse Aggregate Particle Motion

**Figure 12:**
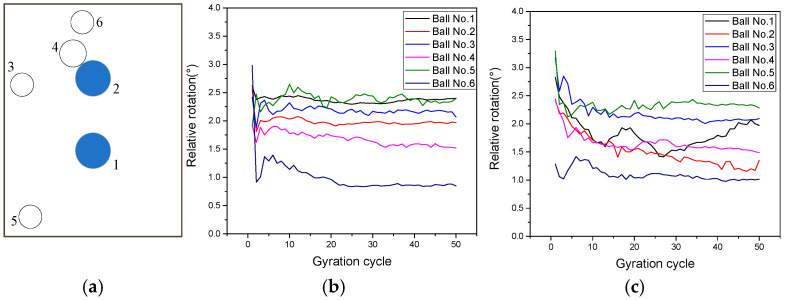
Relative rotation of SmartKli and selected coarse aggregate simulation balls: (**a**) the distribution of balls (**b**) original DEM, (**c**) optimized DEM.

The above discussions showed that the rotation of the SmartKli simulation balls was improved in the optimized model, which could be attributed to the direct data fusion of its measured and predicted values. The optimization effect of other coarse aggregate particle rotations also needs to be evaluated because the asphalt mixture structure is supported by a certain amount of coarse aggregates, and their motions would significantly affect the mixture’s compaction. Thus, in this section, four coarse aggregate particles (numbers 3 to 6) were selected to represent different spatial locations in the specimen; among the balls, particle No. 4 was close to SmartKli ball No. 2. The locations corresponding to the two SmartKli simulation balls (Numbers 1 and 2 in blue) from the front view perspective are plotted in Figure 12. The diameter of the coarse aggregate balls Numbers 3 to 6 was 13.9 mm, 16.9 mm, 15.2 mm, and 13.4 mm, respectively. The particles’ relative rotation in the original DEM and optimized DEM is also shown in Figure 12. The results demonstrated that the changing trend in the coarse aggregate particles’ relative rotation also improved. They did not show a sharp decreasing trend at the beginning of compaction but decreased gradually in the early compaction stage before stabilization. However, the effects of the optimization on the coarse aggregate particles’ stabilized relative rotation were not obvious, which could be due to the limited amount of SmartKli particles (only two in this study). Also, particle No. 6 was affected by the compressing plate, and thus, its rotation responses were not evidently enhanced.

#### 3.2.3. Representativeness of the SmartKli Simulation Ball to General Coarse Aggregate Particles

**Figure 13:**
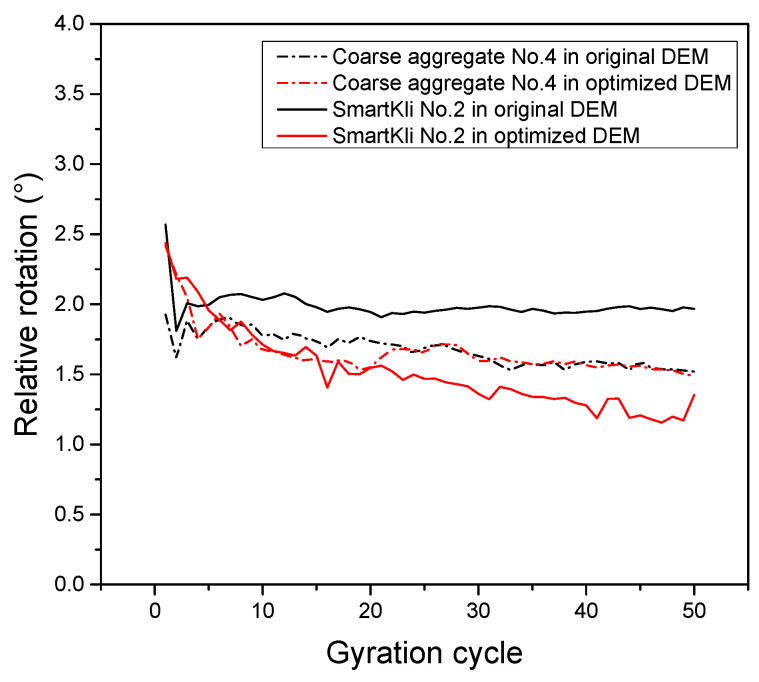
Relative rotation of SmartKli No. 2 and coarse aggregate No. 4 in original and optimized DEM.

It can be seen in Figure 12 that the location of coarse aggregate ball No. 4 was close to SmartKli ball No. 2. To evaluate the representativeness of the SmartKli simulation ball to coarse aggregate particles, the relative rotation of ball No. 2 and No. 4 in the original and optimized DEM models was further plotted in Figure 13. Figure 13 shows that after optimization, the relative rotation of particles in coarse aggregate No. 4 was improved. Moreover, the relative rotation curve of coarse aggregate No. 4 and SmartKli ball No. 2 moved closer to each other. This finding proved that by using the optimized DEM model, the reliability of the SmartKli simulation ball to represent the rotation status of its surrounding coarse aggregates was enhanced, and thus, the SmartKli ball could more accurately reflect an asphalt mixture’s compaction characteristics at the meso-scale. The results demonstrated that the proposed optimization method works effectively in improving the accuracy of coarse aggregate rotation responses obtained in DEM.

Overall, the results suggested that the optimized DEM model has greatly enhanced the accuracy of particle rotation responses during compaction, including both the SmartKli particle and other coarse aggregate particles. Since previous studies indicated the significance of particle relative rotation in characterizing the mixture’s compaction degree [11], the optimization process would greatly help with the utility of the DEM model in predicting an asphalt mixture’s compaction properties.

### 3.3. Evaluation of the Optimized DEM Model in Predicting the Compaction Status

The relative rotation curve of particles during gyratory compaction can be used to characterize the compaction process from the perspective of a mixture’s internal structure. The transition cycle was found to be fundamentally related to the mixture’s packing features at the particle scale [11]. The percent relative rotation in each cycle is calculated as the ratio of the minimum relative rotation at the end of compaction to the relative rotation at cycle i. It could indicate the compaction status at the particulate scale [11].

**Figure 14:**
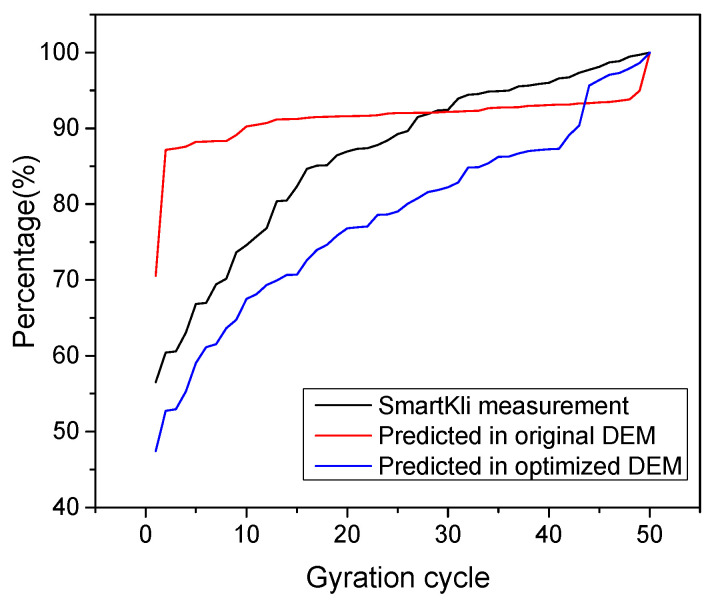
%Relative rotation curve as predicted in DEM models and measured by SmartKli sensor.

In order to evaluate the effectiveness of using the optimized DEM model to characterize the compaction process, the transition cycle N and percent relative rotation calculated in different situations (original DEM, optimized DEM, and laboratory test) are summarized in Table 5 and Figure 14. The quantitative results in Table 5 demonstrated that, in the original DEM model, the relative rotation would transform to a stable status in only three cycles, which was not reasonable because it was only at the very beginning of the compaction process, and the particles still needed more cycles to rotate and adjust their positions. This phenomenon could be due to the assumptions adopted in the modeling process that differentiated the simulation model from the reality conditions. After the DEM model was optimized, the transition cycle of the upper and lower SmartKli increased to 23 and 12, respectively, which moved much closer to the SmartKli sensing results. Compared with the SmartKli sensing results, the transition cycle error predicted in the DEM model decreased from an average of 22 cycles to 7.5 cycles, which greatly enhanced the model’s reliability. As for the percent relative rotation curve in the original DEM model, it had a sharp increasing trend in the first few cycles and did not change much later, which did not conform to the gradual densification process of the asphalt mixture. After optimization, the percent relative rotation curve tended to change gradually.

To summarize, through the optimization method proposed in this study, the particle rotation output in the DEM model improved its reliability and accuracy in predicting the compaction status of the asphalt mixture for gyratory compaction.

## 4. Conclusions and Recommendations

This study made the very first attempt to utilize a type of newly developed smart sensor, SmartKli, to realize real-time data fusion of laboratory measurements with DEM predictions of asphalt mixture SGC compaction. Multiple SmartKlis were settled at different locations in both real and virtual specimens to investigate the particle rotation characteristics and discuss the gyratory compaction mechanism. The effects of the DEM optimization process on the particles’ rotation responses and the reliability of using it to predict the compaction status were further evaluated.

Laboratory test and DEM simulation results showed that the particle’s stabilized relative rotation angle would increase with the decrease in particle height until it reached 2.3°. Particles in the upper location tended to have smaller relative rotation values and needed more gyration cycles to stabilize. This illustrated that in the SGC working mechanism, the upper layer would receive more compactive energy from the top plate and possess a higher compaction degree than the lower layer.

However, the particle rotation responses detected in the laboratory test and DEM simulation were still different due to their different monitoring mechanisms, which demonstrated the necessity of DEM model optimization. Thus, the Kalman filter was used in this study to realize the real-time model optimization process.

The results showed that in the optimized DEM, particle rotation moved closer to the laboratory SmartKli sensing results. The change in the particle relative rotation curve after optimization was located between the measured SmartKli and the original DEM-predicted values. Most importantly, the changing trend of particle relative rotation was improved for both SmartKli and other coarse aggregates. The SmartKli simulation ball could well represent the rotation status of its surrounding coarse aggregates and, thus, could be used to reflect the asphalt mixture’s compaction characteristics.

To summarize, through the optimization method proposed in this study, the particle rotation output in the DEM model improved its reliability and accuracy in predicting the compaction status of an asphalt mixture for gyratory compaction. In further study, the optimized model can be used to accurately observe the phenomena that cannot be achieved by SmartKli sensors or other experiments, such as the 3D distribution of air voids, the development of force chains, the spatial arrangement of aggregate particles, etc.

This paper revealed gyratory compaction characteristics by using multiple SmartKlis in both a laboratory test and DEM simulation. The data fusion of laboratory measurements and DEM prediction values provides a new way to optimize the gyratory compaction DEM model in real time. The particle rotation was optimized in this study because of its significant impact on the mixture’s compaction process. In future studies, particle displacement must also be optimized to comprehensively enhance the simulation precision, especially at the particle scale. Compaction tests with different types of asphalt mixtures are needed to systematically characterize the particle rotational behavior and eventually enhance the reliability and accuracy of the optimized model. In addition, the effects of smart computing parameters on the optimization results need to be further discussed to help researchers decide the most suitable parameters.

## Figures and Tables

**Table 1:** Gradation and mix design information for AC mixture.

Sieve Sizes (mm)	% Passing
19	100
12.5	98
9.5	88
4.75	54
2.36	35
1.18	21
0.6	14
0.3	10
0.15	8
0.075	6.3
Asphalt type	PG 64-22
Asphalt content	5.8%
Anti-strip	0.3%
Design air void	4%
VMA (voids in mineral aggregate)	16%
VFA (voids filled with asphalt)	74.8%

**Table 2:** Denotations of Burger’s model parameters.

	Normal Stiffness	Normal Viscosity	Shear Stiffness	Shear Viscosity
Kelvin	K_kn_	C_kn_	K_ks_	C_ks_
Maxwell	K_mn_	C_mn_	K_ms_	C_ms_

**Table 3:** SGC DEM model parameter.

Parameter	AC
Ball size range (mm)	2.36–19
K_kn_, K_ks_ (N/m)	6.4 × 10^3^, 2.1 × 10^3^
C_kn_, C_ks_ (N/m)	88
K_mn_, K_ms_ (N/m)	54
C_mn_, C_ms_ (N/m)	35
Friction	21
Damping ratio	14
Percentage of simulated asphalt matrix (%)	10
Gyration accelerated scale factor	8

**Table 4:** Transition cycle N in laboratory experiments and DEM simulations.

Transition Cycle N	Upper Particle	Lower Particle
Lab-trial 1	30	20
Lab-trial 2	20	10
DEM-trial 1	3	3
DEM-trial 2	4	3

**Table 5:** Transition cycle predicted in DEM models and measured by SmartKli sensor.

Transition Cycle N	Original DEM	Optimized DEM	SmartKli Sensor
Upper SmartKli	3	23	30
Lower SmartKli	3	12	20

## Data Availability

The data presented in this study are available on request from the corresponding author.

## References

[1] Sadasivam S. (2004). Evaluation of the Effects of Compaction Methods on the Predicted Performance of Superpave Mixtures. Ph.D. Thesis.

[2] Wang X., Shen S., Huang H., Yu S. (2023). Understanding the Role of Particle Rotation in Asphalt Mixture Compaction by Tracking Coarse Aggregate Movement. Constr. Build. Mater..

[3] Wang X., Shen S., Huang H., Zhang Z. (2019). Towards smart compaction: Particle movement characteristics from laboratory to the field. Constr. Build. Mater..

[4] Masad E., Jandhyala V.K., Dasgupta N., Somadevan N., Shashidhar N. (2002). Characterization of air void distribution in asphalt mixes using X-ray computed tomography. J. Mater. Civ. Eng..

[5] Partl M.N., Flisch A., Jnsson M. (2003). Gyratory compaction analysis with computer tomography. Road Mater. Pavement Des..

[6] Sefidmazgi N.R., Tashman L., Bahia H. (2012). Internal structure characterization of asphalt mixtures for rutting performance using imaging analysis. Road Mater. Pavement Des..

[7] Chen J. (2011). Discrete Element Method (DEM) Analyses for Hot-Mix Asphalt (HMA) Mixture Compaction. Ph.D. Thesis.

[8] Chen J., Huang B., Shu X. (2013). Air-void distribution analysis of asphalt mixture using discrete element method. J. Mater. Civ. Eng..

[9] Gong F., Zhou X., You Z., Liu Y., Chen S. (2018). Using discrete element models to track movement of coarse aggregates during compaction of asphalt mixture. Constr. Build. Mater..

[10] Liu W.D., Gong X., Xiong J.P., Zhou S.B. (2020). Analysis of influence of aggregate stiffness on coarse aggregate movement of asphalt mixture. IOP Conf. Ser. Earth Environ. Sci..

[11] Wang X., Shen S., Huang H., Almeida L.C. (2018). Characterization of particle movement in Superpave gyratory compactor at meso-scale using SmartRock sensors. Constr. Build. Mater..

[12] Wang X., Shen S., Huang H. (2021). Meso-Scale Kinematic Responses of Asphalt Mixture in Both Field and Laboratory Compaction. Transp. Res. Rec..

[13] Dan H.-C., Yang D., Zhao L.-H., Wang S.-P., Zhang Z. (2020). Meso-scale study on compaction characteristics of asphalt mixtures in Superpave gyratory compaction using SmartRock sensors. Constr. Build. Mater..

[14] Yu S., Shen S., Steger R., Wang X. (2022). Effect of warm mix asphalt additive on workability of asphalt mixture: From particle perspective. Constr. Build. Mater..

[15] Yu S., Shen S. (2022). Compaction Prediction for Asphalt Mixtures Using Wireless Sensor and Machine Learning Algorithms. IEEE Trans. Intell. Transp. Syst..

[16] Liu Y., Zhou X., You Z., Yao S., Gong F., Wang H. (2017). Discrete element modeling of realistic particle shapes in stone-based mixtures through MATLAB-based imaging process. Constr. Build. Mater..

[17] Xing C., Liu B., Liu H., Zhang L., Xu H., Tan Y. (2024). Topological characterization and typical topologies of disruption aggregates in asphalt mixture. J. Mater. Civ. Eng..

[18] Komaragiri S., Gigliotti A., Bhasin A. (2021). Feasibility of using a physics engine to virtually compact asphalt mixtures in a gyratory compactor. Constr. Build. Mater..

[19] Yu H., Shen S. (2012). Impact of aggregate packing on dynamic modulus of hot mix asphalt mixtures using three-dimensional discrete element method. Constr. Build. Mater..

[20] Zhou X., Chen S., Ge D., Jin D., You Z. (2020). Investigation of asphalt mixture internal structure consistency in accelerated discrete element models. Constr. Build. Mater..

[21] Tan Y., Liang Z., Xu H., Xing C. (2022). Research on Rutting Deformation Monitoring Method Based on Intelligent Aggregate. IEEE Trans. Intell. Transp. Syst..

[22] Tan Y., Liang Z., Xu H., Xing C. (2022). Internal deformation monitoring of granular material using intelligent aggregate. Autom. Constr..

[23] Zhang C., Ildefonzo D.G., Shen S., Wang L., Huang H. (2023). Implementation of ensemble Artificial Neural Network and MEMS wireless sensors for In-Situ asphalt mixture dynamic modulus prediction. Constr. Build. Mater..

[24] Zhang C., Shen S., Huang H., Wang L. (2021). Estimation of the Vehicle Speed Using Cross-Correlation Algorithms and MEMS Wireless Sensors. Sensors.

[25] Shi B., Shen S., Liu L., Wang X. (2021). Estimation of vehicle speed from pavement stress responses using wireless sensors. J. Transp. Eng. Part B Pavements.

[26] Itasca-Consulting-Group PFC5.0 Suite Documentation 2014. Minneapolis, Minnesota 55415. https://docs.itascacg.com/pfc600/common/docproject/source/manual/program_guide/program_guide.html?node66.

[27] Shen S., Yu H. (2011). Characterize packing of aggregate particles for paving materials: Particle size impact. Constr. Build. Mater..

[28] Liu S., Huang H., Qiu T., Shen S. (2018). Sensing mechanism and real-time computing for granular materials. J. Comput. Civ. Eng..

[29] Kalman R.E. (1960). A new approach to linear filtering and predicted problems. J. Fluids Eng..

[30] Wang X., Wang Z., Luo X. Characterization of asphalt mixture compaction at particle-scale using discrete element model optimized by real-time smartrock sensing data (No. TRBAM-25-03625). Proceedings of the Transportation Research Board 104th Annual Meeting Compendium of Papers.

